# Oxamate, but Not Selective Targeting of LDH-A, Inhibits Medulloblastoma Cell Glycolysis, Growth and Motility

**DOI:** 10.3390/brainsci8040056

**Published:** 2018-03-30

**Authors:** Cara J. Valvona, Helen L. Fillmore

**Affiliations:** Cellular & Molecular Neuro-oncology Research Group, University of Portsmouth, School of Pharmacy & Biomedical Sciences, Portsmouth PO1 2DT, UK; Cara.valvona@port.ac.uk

**Keywords:** LDHA, lactate dehydrogenase, medulloblastoma, aerobic glycolysis, Warburg effect, oxamate

## Abstract

Medulloblastoma is the most common malignant paediatric brain tumour and current therapies often leave patients with severe neurological disabilities. Four major molecular groups of medulloblastoma have been identified (Wnt, Shh, Group 3 and Group 4), which include additional, recently defined subgroups with different prognosis and genetic characteristics. Lactate dehydrogenase A (LDHA) is a key enzyme in the aerobic glycolysis pathway, an abnormal metabolic pathway commonly observed in cancers, associated with tumour progression and metastasis. Studies indicate MBs have a glycolytic phenotype; however, LDHA has not yet been explored as a therapeutic target for medulloblastoma. LDHA expression was examined in medulloblastoma subgroups and cell lines. The effects of LDHA inhibition by oxamate or LDHA siRNA on medulloblastoma cell line metabolism, migration and proliferation were examined. LDHA was significantly overexpressed in Group 3 and Wnt MBs compared to non-neoplastic cerebellum. Furthermore, we found that oxamate significantly attenuated glycolysis, proliferation and motility in medulloblastoma cell lines, but LDHA siRNA did not. We established that aerobic glycolysis is a potential therapeutic target for medulloblastoma, but broader LDH inhibition (LDHA, B, and C) may be more appropriate than LDHA inhibition alone.

## 1. Introduction

Brain and other central nervous system (CNS) tumours are the second largest cause of cancer-related deaths in children, after leukaemia. Medulloblastomas (MBs) account for 20% of paediatric brain tumours, making them the most common type of solid, malignant childhood brain tumour [1]. MB manifests in the cerebellum and has a tendency to spread to the spinal cord. Four main subgroups of MB have been identified and are now recognised by the World Health Organisation (WHO): as Wnt-activated, Shh-activated TP53 wild-type, Shh-activated TP53 mutant and non-Wnt/non-Shh, the latter of which has been subdivided into “Group 3” and “Group 4” [2,3]. Recent studies reveal even further divisions [4]. Each group has disparate features including demographics, clinical outcomes, characteristic genetic abnormalities and rates of metastasis. Although the current five-year event-free survival is >80% for low-risk children, and 60–70% for high-risk children [5], the quality of life of individuals after treatment is often significantly reduced.

Regardless of oxygen availability, cancer cells commonly use aerobic glycolysis for ATP production; this is known as the Warburg effect [6]. Pyruvate is diverted away from the mitochondria, where it normally undergoes oxidative phosphorylation (OXPHOS) to generate ATP. Instead, it is converted to lactate by LDHA and nicotinamide adenine dinucleotide (NAD+) is regenerated from (NAD)H in the process [7]. NAD+ is required by glyceraldehyde 3-phosphate dehydrogenase to maintain glycolysis and ATP production. This reaction also occurs occasionally in some specialised non-neoplastic cells during rapid proliferation and other non-neoplastic cells when oxygen is unavailable. Nevertheless, as this is the preferential method of ATP production used by cancer cells, it is an attractive target for cancer therapies. We previously reviewed the regulation and function of LDHA and its therapeutic potential in brain tumours [8]. 

Mouse models have shown that during normal development of the cerebellum, cerebellar granule neural precursors (CGNPs) are stimulated by Shh to undergo aerobic glycolysis. Shh and phosphoinositide 3-kinase signalling has been shown to stimulate aerobic glycolysis in CGNPs in a hexokinase-2 (HK2)-dependent manner [9]. Furthermore, inhibition of aerobic glycolysis by deleting HK2 in Shh MB mouse models reduced MB malignancy by promoting differentiation and reducing proliferation [9]. Shh MBs also exhibit elevated lipid synthesis implicated in aerobic glycolysis [10]. 

Using magnetic resonance spectroscopy and 18fluorodeoxyglucose positron emission tomography, MBs in patients have been shown to have a glycolytic metabolic phenotype and a recent study has shown elevated lactate levels were associated with a Group 3/4 subgroup [11,12]. MBs also have elevated expression of monocarboxylate transporter 1 (MCT1), which transports lactate and pyruvate across the plasma membrane, also indicating a glycolytic phenotype [13]. Moreover MCT1 is inhibited by miR-124, which is commonly downregulated in MB, and ectopic expression of miR-124 has been shown to inhibit MB proliferation by blocking cell cycle progression at G1. 

Cellular metabolism can be influenced by many pathways, including several implicated in MB. The Wnt signalling network has been shown to increase glycolysis and decrease oxidative phosphorylation [14,15]. C-Myc, which is overexpressed in Group 3 MB and activated downstream of Wnt signalling pathways, is also known to promote glycolysis in many cancers [2,16] and upregulates LDHA expression [17]. Although MB is made up of four distinct subgroups, together these studies indicate that aerobic glycolysis is a common feature of most if not all MBs.

LDHA has not yet been explored as a therapeutic target for MB. We used oxamate, a structural analogue of pyruvate, which competes with pyruvate to inhibit LDHA activity (not expression) [18] and LDHA siRNA to investigate the therapeutic potential of targeting LDHA in MB. We hypothesised that LDHA inhibition would result in a decrease in lactate concentrations and a change from a glycolytic to an oxidative phosphorylation metabolic phenotype, leading to decreased medulloblastoma, proliferation and motility.

## 2. Materials and Methods

**Cell culture:** Human MB cell lines UW402, Res256 (Dr. John Silber, University of Washington, Seattle, WA, USA) were cultured in Dulbecco’s Modified Eagle’s medium (Gibco, Paisley, UK), 10% FBS. Human MB cell line DAOY (ATCC, London, UK) was cultured in Eagle’s minimum essential medium (ATCC), 10% FBS. Cells were maintained in a 37 °C, 95% air, 5% CO_2_ in a humidified incubator (NUAIRE, Caerphilly, UK). Cells were routinely screened for mycoplasma using the MycoAlert™ mycoplasma detection kit (Lonza, London, UK).

**Analysis of patient data:** Gene expression datasets were obtained and analysed using the R2 genomics and visualisation platform (http://r2.amc.nl) [19] LDHA expression was analysed from a selection of CNS tissues available from the Normal Various-Roth-504–MAS5.0–u133p2 dataset and in MBs using the Tumor Medulloblastoma-Gilbertson-76–MAS5.0–u133p2 dataset. 

**Western blot:** Cells were lysed with M-PER^®^ (Thermo Scientific, Basingstoke, UK) and Halt™ protease and phosphatase inhibitor cocktail (Thermo Scientific, Basingstoke, UK, 1:100). 50 µg of protein was separated by SDS-PAGE in Tris-Glycine 4–20% gels (Thermo Scientific, Basingstoke UK) and transferred onto Immun-Blot^®^ PVDF membrane (Bio-Rad, Oxford, UK). Blots were incubated overnight at 4 °C in 5% milk blocking buffer containing LDHA (Novus Biologicals, Oxford, UK) or Cyclophilin A antibody (Abcam, Cambridge, UK). The blots were washed in TBST and incubated with Goat anti-rabbit IgG horseradish peroxidase antibody (Promega, Southampton, UK) for 1 h at room temp. The blots were incubated with Luminata™ Forte Western HRP Substrate (Merck Millipore, Watford, UK) and imaged using the G-Box chemiluminescent imaging system (Syngene, Cambridge, UK). Semi-quantitative downstream analysis of protein expression was performed using Image J software.

**Flow Cytometry:** Cells were harvested and centrifuged at 300 RCF at 4 °C, the supernatant removed and pellet washed in PBS (Gibco, Poole, UK). The pellet was re-suspended in of BD cytofix/cytoperm™ fixation and permeabilisation solution (BD Biosciences, Oxford, UK), and incubated for 20 min at 4 °C in the dark. The cells were washed with wash buffer (PBS with 0.2% saponin, and 1% goat serum) and re-suspended with LDHA antibody (Novus Biologicals) and incubated for 30 min at 4 °C. Cells were washed with wash buffer and re-suspended with goat anti-rabbit IgG Alexa fluor 488 (Life Technologies, Warrington, UK), and incubated for 15 min at 4 °C. Cells were washed with wash buffer and re-suspended in staining buffer (PBS with 1% goat serum and 0.09% *w*/*v* sodium azide pH to 7.4–7.6) and 20,000 events from each sample analysed using a BD FACSCalibur™. Acquisition and analysis were performed using CellQuestPro software to determine cell line expression of antigen.

**Oxamate:** Cells were seeded and left to adhere overnight before treatment with oxamate (0 h). Oxamate (Sigma Aldrich, Poole, UK) stock was prepared at 0.5 M concentrations in dH_2_O and filtered, using a Millex^®^-GP 22µm filter unit (Merck Millipore, Watford, UK).

**Small interfering RNA (siRNA) knockdown:** Stock LDHA Silencer^®^ select pre-designed siRNA (Ambion, Basingstoke, UK, 4392420) and Silencer^®^ select Negative control #1 siRNA (Ambion, UK) in nuclease-free water (Ambion, UK) were diluted in Opti-MEM (Gibco, UK). The siRNA stock was mixed with jetPRIME^®^ buffer and jetPRIME^®^ reagent (Polyplus transfection^®^, Source Bioscience Life Sciences, Nottingham, UK) according to the manufacturer’s instructions. 15 nmol siRNA was added to 70,000 cells in 12-well plates and the media changed after 24 h. LDHA knockdown was verified by Western blots parallel to every functional siRNA experiment.

**Lactate assay:** Lactate concentrations were measured using a lactate assay kit (MAK064, Sigma Aldrich, UK) according to the manufacturer’s instructions. Cells were lysed and protein concentration determined by BCA. 20 µL of lysates were analysed at 570 nm using POLARstar OPTIMA plate reader (BMG Labtech, Aylesbury, UK).

**Glycolysis stress test and Mito stress test:** Glycolysis and mitochondrial activity was measured using the Seahorse Bioanalyser 96XF with Seahorse Bioanalyser Glycolysis stress test kit and Mito stress test kit according to the manufacturer’s instructions respectively. Cells were treated with oxamate 24 h prior to analysis and data analysis was conducted using Wave software.

**Trypan blue exclusion assay:** Cells were harvested from six-well plates, transferred into Vi-Cell cups and counted using the Vi-Cell^®^ (BeckMan Coulter, High Wycombe, UK) automated trypan blue dye exclusion assay. Seeding density was optimised for each cell line to ensure that no more than 85% confluence was achieved by the 72 h time point in the control wells.

**Cell cycle analysis:** Cells were harvested from six-well plates and centrifuged at 500 RCF at room temperature, the supernatant discarded then washed and re-suspended in PBS (Gibco, UK). The cells were fixed with cold 70% ethanol and stored at 4 °C for seven days. The ethanol-suspended cells were centrifuged at 800 RCF at 4 °C and the supernatant removed. The cells were washed with PBS and re-suspended in Chemometec Solution 3 (1 µg/mL DAPI, 0.1% triton x-100 in PBS). 10 µL of each sample was loaded into a chamber of a NC-Slide A8™ and >10,000 cells analysed using the NucleoCounter^®^ NC-3000.

**Live cell imaging:** A scratch was made through a monolayer of cells in a 24-well plate using a pipette tip. The media was removed and replaced with 500 µL of appropriate media (with/without oxamate) and secured into a Ziess Axiovert 200 M microscope stage enclosed in a temperature (tempcontrol 37-2 digital, Meyer instruments, Houston, TX, USA), CO_2_ and oxygen (Bold line, Okolab, Indigo Scientific, Baldock, UK) controlled incubator set to 37 °C, 95% air, 5% CO_2_. Images were taken every 30 minutes using Volocity^®^ acquisition software (PerkinElmer, Chiltern, UK). The area of the gap was measured using T Scratch software developed by the Koumoutsakos group (CSE Lab, Zürich, Switzerland), at ETH Zürich [20]. 10 cells from the edge of each scratch were tracked manually for the first 24 h using Volocity^®^ analysis software (PerkinElmer, Chiltern, UK). 

**Statistics:** All experiments were completed together and independently in triplicate. All statistical analysis was carried out using GraphPad Prism 6 software (6.0, GraphPad Software, San Diego, CA, USA). *p*-values less than 0.05 were considered significant. *t* tests were used to analyse the WB and FC data. Ordinary one-way ANOVA was used to analyse the R2 database genomic data and to analyse 24 h of cell tracking data. Two-way ANOVA was used for all other experiments.

## 3. Results

### 3.1. LDHA Expression in MB

**Figure 1:**
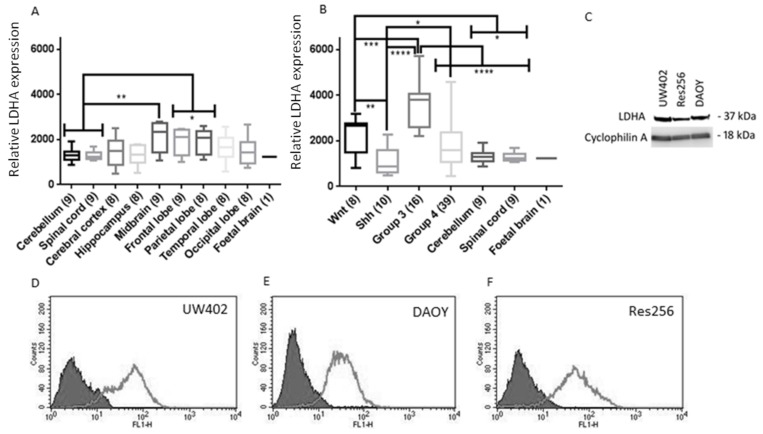

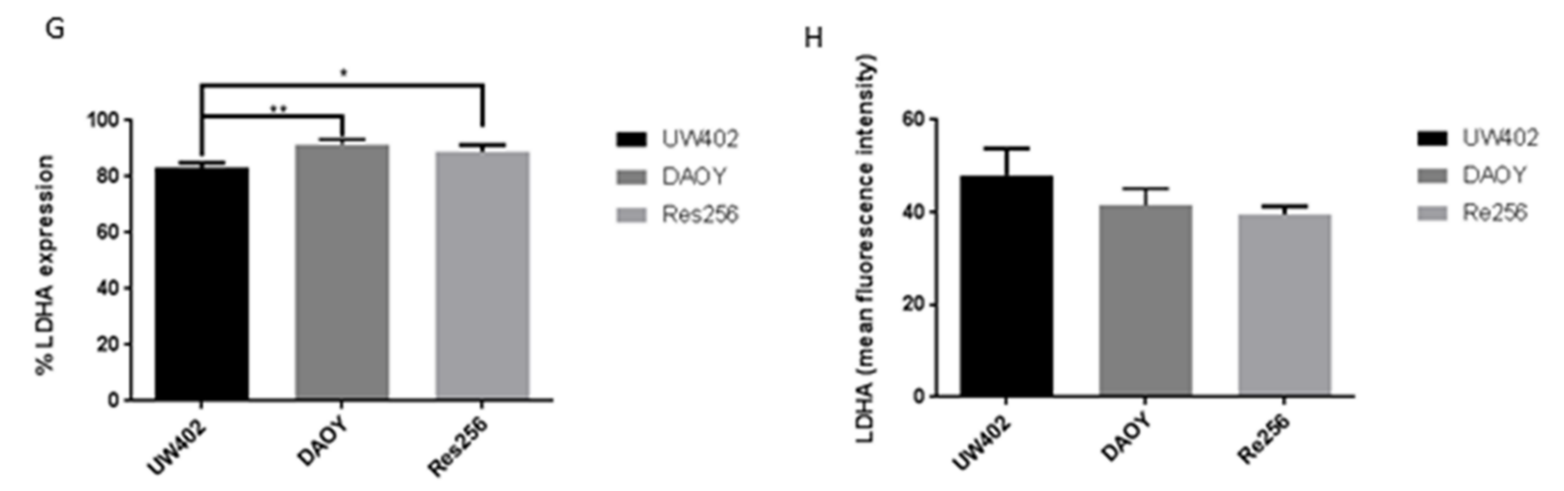
LDHA expression in MB and non-neoplastic tissue and cell lines. (**A**,**B**) LDHA expression in CNS tissues from data extracted using the R2 data base. The number of samples per tissue is stated in parentheses. Graphs show box plots (highest value, lowest value, upper quartile, lower quartile, interquartile range and median). (**A**) LDHA expression in non-neoplastic CNS tissues. LDHA expression in cerebellum and CNS were compared to every other CNS tissue; (**B**) LDHA expression in a range of non-neoplastic CNS tissues and MB subgroups. LDHA expression was compared between MB subgroups and compared to normal cerebellum and spinal cord; (**C**) Representative Western blot of lysates from MB cell lines UW402, Res256 and DAOY. 50µg of protein was loaded into each well and the blot was probed for LDHA (37 kDa) and cyclophillin A (18 kDa) as a loading control; (**D**–**I**) Flow cytometry analysis of LDHA expression in MB cell lines; solid grey = negative control, light grey line = LDHA expression (**D**) UW402; (**E**) DAOY; (**F**) and Res256; (**G**) Percentage of cell populations expressing LDHA. (**H**) Mean LDHA expression (fluorescence intensity). Graphs show mean with SEM. Not significant *p* > 0.05, * *p* ≤ 0.05, ** *p* ≤ 0.01, *** *p* ≤ 0.001, **** *p* ≤ 0.0001.

Analyses of LDHA mRNA levels were conducted using publicly available datasets (Figure 1A,B). Non-neoplastic cerebellum expressed low levels of LDHA compared to other non-neoplastic CNS tissue, and significantly less than the midbrain (*p* ≤ 0.01), parietal lobe and frontal lobe (*p* ≤ 0.05). The dataset used does not specify the age range of the patient samples; however, this finding correlates with previous studies that used magnetic resonance imaging and positron emission tomography scans in neurologically normal young adults. One study found that aerobic glycolysis was significantly elevated in the medial and lateral parietal and prefrontal cortices; however, the cerebellum had significantly lower levels of aerobic glycolysis than the rest of the brain [21]. There are limited data available on the expression of LDHA in the human cerebellum during prenatal and postnatal neurogenesis, but it may correlate with other genes associated with glycolysis that have been investigated. Goyal et al. [22] found that during the first few months of gestation, an increase in the expression of 116 glycolytic genes occurs in parallel across the brain. Interestingly, at mid-gestation the expression of these 116 glycolytic genes falls sharply in the cerebellum but stabilizes in the diencephalon, and continues to increase in the striatum and cerebral cortex. 

We also examined the differences in LDHA expression between the four MB subgroups and found that LDHA expression was as follows; Group 3 (*N* = 16) > Wnt (*N* = 8) > Group 4 (*N* = 39) > Shh (*N* = 10) Group 3 and Wnt MBs also expressed significantly more LDHA than non-neoplastic cerebellum (*N* = 9) and spinal cord tissue (*N* = 9) (*p* ≤ 0.0001 and *p* ≤ 0.05 respectively). Considering that previous studies have shown that Shh promotes aerobic glycolysis [9,23] it was surprising that Shh MBs had lower mean LDHA expression than non-neoplastic cerebellum and spinal cord tissue. As C-myc is a transcription factor for LDHA [17], it is not surprising that Group 3 and Wnt MBs express significantly higher levels of LDHA than non-neoplastic cerebellum and spinal cord, as Group 3 is known to overexpress c-Myc and c-Myc is also a downstream target of the Wnt signalling pathway. Group 3 is the most metastatic subgroup of MB and carries the poorest prognosis; these features are also commonly associated with LDHA and lactate concentrations in other tumours [24]. Group 3 MBs expressed significantly higher levels of LDHA than all the other MB subgroups indicating that LDHA could be a promising therapeutic target specifically against Group 3 MBs. LDHA was also abundantly expressed in MB cell lines (Figure 1C–H).

### 3.2. Oxamate Attenuates MB Proliferation

In order to reveal the potential therapeutic benefits of targeting LDHA in MB, LDHA activity (not expression) was inhibited by oxamate and the downstream effects on MB proliferation, metabolism and motility examined.

**Figure 2:**
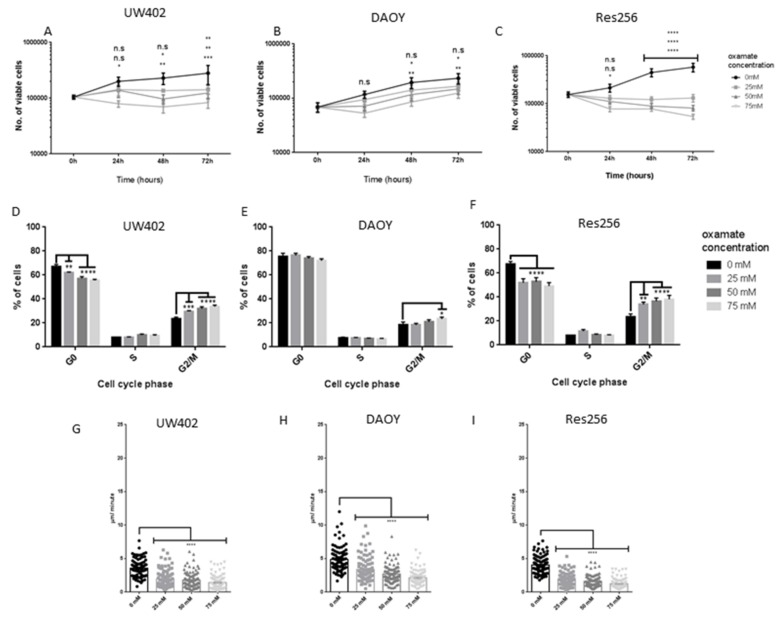
Effects of oxamate on MB proliferation and motility. MB cell lines were grown in the presence of 0 mM, 25 mM, 50 mM or 75 mM oxamate. (**A**–**C**) Number of viable cells 0–72 h after treatment counted by Vi-Cell automated cell counter (**A**) UW402, (**B**) DAOY and (**C**) Res256; (**D**–**F**) Mean percentage of cells in G0, S and G2/M phases of the cell cycle 48 h after treatment (**D**) UW402, (**E**) DAOY, (**F**) Res256; (**G**–**I**) Average velocity (µm/second) of individual (**G**) UW402, (**H**) DAOY and (**I**) Res256 cells over 24 h. Cells were imaged every 30 minutes and tracked manually using Volocity software. Graphs show mean with SEM. Oxamate treated samples were compared to the untreated control at each time point, not significant *p* > 0.05, * *p* ≤ 0.05, ** *p* ≤ 0.01, *** *p* ≤ 0.001, **** *p* ≤ 0.0001.

As shown by previous studies in other cell lines [25,26,27], we found that oxamate significantly attenuated the number of viable MB cells in a time- and concentration-dependent manner, the Res256 cell line was the most sensitive and DAOY the least sensitive (Figure 2A–C). By inhibiting LDHA and aerobic glycolysis, oxamate could reduce the rate at which ATP can be generated and therefore the rate at which MB cells can proliferate. It is possible that because Res256 had a lower expression of LDHA, lower concentrations of oxamate were able to have a more significant effect. Further investigations indicated that oxamate treatments significantly reduced the percentage of MB cells in G0/G1 and significantly increased the percentage of MB cells in G2/M in a concentration dependent manner (Figure 2D–F). Res256 and UW402 cells were similarly sensitive whereas DAOY cells were somewhat less sensitive. Together, these experiments suggest that oxamate caused a G2/M cell cycle arrest in MB cells, as reported previously in nasopharyngeal carcinoma cells [25]. However, further investigations are required to determine why DAOY cells are less affected by oxamate, one possibility is that they are better adapted to switching from aerobic glycolysis to OXPHOS.

### 3.3. Oxamate Inhibits MB Cell Speed of Migration

Approximately 30% of MBs are metastatic at diagnosis and are associated with a poorer prognosis [28] and previous studies in other cancers have shown that lactate is a prognostic factor for metastasis [24,29,30]. Using live cell imaging, we found that oxamate significantly inhibited MB cell migration (*p* ≤ 0.0001) (Figure 2G–I). Together these studies show that by inhibiting LDHA activity and lactate production, MB motility is significantly reduced, a finding that could be of great benefit when developing therapies to reduce the devastatingly metastatic nature of the disease.

### 3.4. Oxamate Inhibits MB Aerobic Glycolysis and Promotes OXPHOS

**Figure 3:**
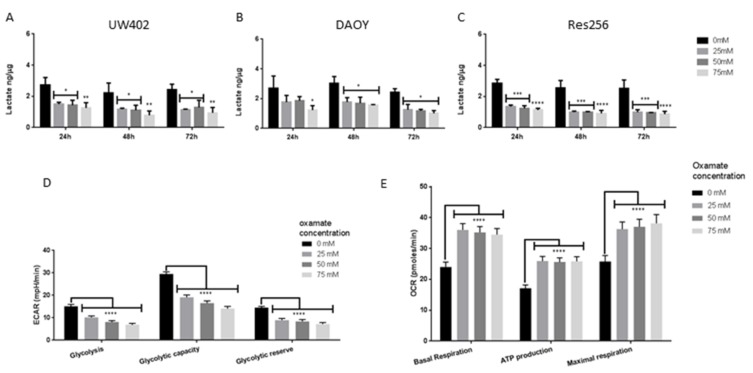
Effects of oxamate on MB metabolism. MB cell lines were grown in the presence of 0 mM, 25 mM, 50 mM or 75 mM oxamate. (**A**–**C**) Lactate concentrations of lysates prepared from (**A**) UW402, (**B**) DAOY, and (**C**) Res256 cells treated for 24–72 h. Lactate concentrations were normalised to the protein concentrations of each sample. (**D**) ECAR (mpH/min) of UW402 cells treated for 24 h. Glycolysis, glycolytic capacity and glycolytic reserve calculated from changes in ECAR with the addition of glucose, oligomycin and 2-DG. (**E**) Oxygen consumption rate (pmoles/min) of UW402 cells treated for 24 h. Basal respiration, ATP production and maximal respiration calculated from changes in OCR with the addition of oligomycin, FCCP, rotenone and antimycin A. Oxamate-treated samples were compared to the untreated control at each time point. Graphs show mean with SEM, not significant *p* > 0.05, * *p* ≤ 0.05, ** *p* ≤ 0.01, *** *p* ≤ 0.001, **** *p* ≤ 0.0001.

The abundant expression of LDHA and lactate in the MB cell lines suggests they, like most cancers, have a glycolytic phenotype [6]. As shown previously in other cell lines [25,26,27], we found that oxamate significantly inhibited lactate concentrations in every cell line (Figure 3A–C), indicating successful inhibition of LDHA activity, lactate production and the glycolytic phenotype. 

Although all three MB cell lines showed high levels of LDHA expression, the UW402 MB cell line was chosen for examination in more detail as it is known to have isochromosome 17q, which is typical of Group 3 and Group 4 MBs, the most metastatic MBs that carry the poorest prognosis [31]. DAOY cells have been molecularly subgrouped as Shh MB [32] and Res256 cells have not been subgrouped, to our knowledge. After 24 h all oxamate treatments significantly reduced UW402 cell glycolysis, glycolytic capacity and glycolytic reserve (*p* ≤ 0.0001) (Figure 3D). Furthermore, all oxamate treatments caused a significant increase in UW402 cell basal respiration, ATP production and maximal respiration (*p* ≤ 0.0001), suggesting an increase in OXPHOS metabolism (Figure 3E). Together these studies confirm that oxamate significantly altered MB metabolism, reduced LDHA activity, aerobic glycolysis and lactate production, and increased OXPHOS in UW402 cells.

### 3.5. Oxamate and LDHA as a Therapeutic Target for MB

**Figure 4:**
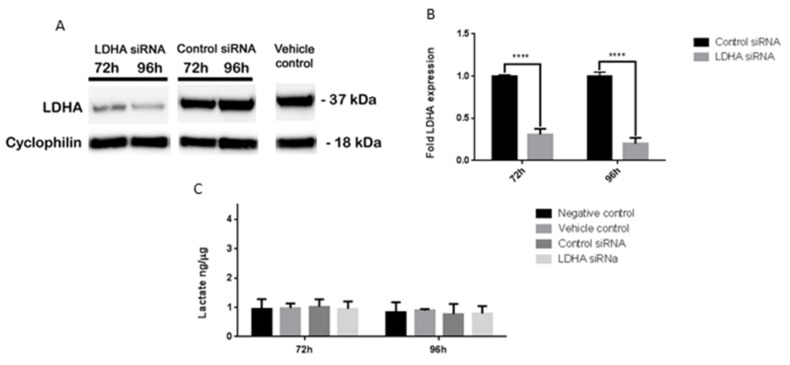
Effects of LDHA siRNA on UW402. UW402 cells were untreated, treated with vehicle control (jet prime transfection reagent), LDHA siRNA and jet prime transfection reagent, or control siRNA and jet prime transfection reagent. (**A**,**B**) Western blot of lysates prepared from UW402 cells 72–96 h after transfection. 25µg of protein was loaded into each well and the blot was probed for LDHA (37 kDa) and cyclophillin A (18 kDa) as a loading control. (**A**) Western blot bands; (**B**) Semiquantitative analysis of LDHA expression using image J software. LDHA expression was normalised to Cyclophilin expression. LDHA expression is represented as a fold change from control siRNA LDHA expression at each time point; (**C**) Lactate concentrations of lysates prepared from UW402 cells 72–96 h after transfection. Lactate concentrations were normalised to the protein concentrations of each sample. Graphs show mean with SEM. All samples were compared to each other at each time point, **** *p* ≤ 0.0001.

We have shown that oxamate significantly inhibits MB lactate production, aerobic glycolysis, proliferation and motility and also upregulates OXPHOS. Although the data presented here, and by others, using oxamate and LDHA to target cancer are promising, unfortunately the concentration of oxamate required to have significant effects is far too high to be considered for clinical use. As more suitable LDHA targeted therapies are in development, we wanted to corroborate our findings with LDHA-targeted siRNA (Figure 4).

### 3.6. LDHA siRNA Does Not Inhibit MB Viability, Cell Migration or Metabolism

We found that >80% knockdown of LDHA expression did not have any significant downstream effects on MB cell metabolism, growth or motility (data not shown). However, previous studies in other cell lines that had also not achieved a 100% knockdown of LDHA found it caused a significant reduction in tumour cell migration, growth and maintenance, similar to the results we saw with oxamate [33,34,35]. 

It is possible that as LDHA was not completely knocked down in the MB cells, the remaining LDHA was able to compensate for the loss. Another possibility is that other LDH family members were able to compensate when LDHA was knocked down. LDHA and LDHB can form homo- or heterotetramers that have identical active site regions [8,36], so it is likely that oxamate has inhibitory effects on all five LDH tetramers and that is why it was more effective than LDHA siRNA. 

**Figure 5:**
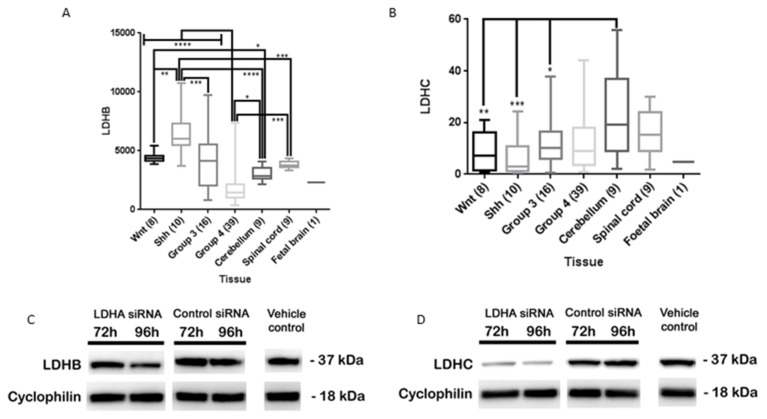
Effects of LDHA siRNA on UW402 LDHB and LDHC expression. (**A**,**B**) Expression of (**A**) LDHB and (**B**) LDHC in MB subgroups, non-neoplastic cerebellum, spinal cord and foetal brain. The number of samples per tissue is stated in brackets. Graphs show box plots (highest value, lowest value, upper quartile, lower quartile, interquartile range and median). Not significant *p* > 0.05, * *p* ≤ 0.05, ** *p* ≤ 0.01, *** *p* ≤ 0.001, **** *p* ≤ 0.0001; (**C**,**D**) Western blots of lysates prepared from UW402 cells treated with jet prime transfection reagent (vehicle control), UW402 cells treated with LDHA siRNA and jet prime transfection reagent and UW402 cells treated with control siRNA and jet prime transfection reagent 72–96 h after transfection. 25 µg of protein was loaded into each well and the blot was probed for C) LDHB or D) LDHC (37 kDa) and cyclophillin A (18 kDa) as a loading control.

Analysis of LDHB gene expression using the R2 genomics and visualisation platform revealed that the mean expression of LDHB within the MB subgroups was as follows; Shh > Wnt > Group 3 > Group 4 (Figure 5A). Shh MBs had significantly higher LDHB expression than the other MB subgroups. As previous studies have shown Shh MBs have a glycolytic phenotype and we found that they express the least amount of LDHA out of the MB subgroups, it is possible that LDHB is undertaking the role normally expected of LDHA. There was no significant difference in LDHC expression between the MB subgroups (Figure 5B). Furthermore, the Wnt, Shh and Group 3 subgroups expressed significantly less LDHC than the cerebellum.

Previous LDHA knockdown studies that show tumour attenuation have not verified the knockdown status of other LDH family members [33,34,35,37]. In our study we found that lysates treated with LDHA siRNA also had marked LDHC knockdown but LDHB expression remained relatively unchanged (Figure 5C,D). Therefore, our results suggest that the attenuation of MB aerobic glycolysis, motility, viability, proliferation and lactate production may require LDHB inhibition as well as LDHA inhibition and possibly LDHC inhibition. However, further extensive studies based on the foundation of these results are necessary to confirm this hypothesis.

## 4. Discussion

The high glucose requirement of MB cells and other tumours makes them vulnerable to therapies that target aerobic glycolysis. We have shown that LDHA was significantly elevated in Group 3 and Wnt MBs and LDHB was significantly upregulated in Shh MBs compared to non-neoplastic cerebellar tissue. We found that oxamate significantly attenuates MB aerobic glycolysis, proliferation and motility however, unlike studies in other tumours; our LDHA knockdown studies did not result in the same functional observations. In our study, we also explored the impact of LDHA siRNA on the other LDH family members and documented that LDHA siRNA knocked down LDHC considerably as well as LDHA, but not LDHB. One explanation for the disparity is that other LDH family members may have been attenuated in other experiments as well but the impact of LDHA knockdown on all LDH family members is not routinely assessed. In addition, our data were obtained from using established MB cell lines in vitro; therefore, additional experiments in more complex models that better represent in vivo micro-environments are needed.

## 5. Conclusions

Further investigations will be critical as LDHA may prove to be an inadequate target for MB and a broader LDH family inhibitor that also targets LDHB may be more appropriate to exploit this weakness whilst limiting damage to non-neoplastic cells. In future studies it would be of interest to explore the knockdown of LDHA, LDHB and LDHC, as well as the use of broad LDH inhibitors in MB mouse models [38,39].
